# Electric Field Characteristics and an Electrostatic Detection Method for Small UAVs Based on Triboelectric Charging

**DOI:** 10.3390/s26217055

**Published:** 2026-11-08

**Authors:** Xinran Liu, Zhiyong Zhang, Manyu Wang, Pengfei Li

**Affiliations:** School of Mechatronics Engineering, Beijing Institute of Technology, Beijing 100081, China; lxr1142180366@126.com (X.L.); zhangzhiy152@163.com (Z.Z.); manyuw116@163.com (M.W.)

**Keywords:** electrostatic detection, triboelectric charging, small UAV, electric-field measurement, target characteristics, multiphysics coupling

## Abstract

**Highlights:**

**What are the main findings?**

- A theoretical triboelectric charging model for UAVs was established, and a high-speed airflow impact experiment was used to calibrate its condition-dependent charge-transfer parameter between the tested airflow and surface material.
- Multiphysics simulations characterized the electrostatic near field of a simplified small-UAV model; the maximum electric-field strength along the nominal 10 m encounter trajectory was 415 V/m.

**What are the implications of the main findings?**

- The electrostatic field generated by triboelectric charging can provide a physical-field signature for complementary passive detection of small UAVs.
- The model provides a framework for analyzing UAV electrostatic characteristics under specified vehicle configurations and operating conditions.

**Abstract:**

The widespread use of small unmanned aerial vehicles (UAVs) has increased the need for reliable short-range detection in complex environments. Interactions between the vehicle surface, air, and suspended particles can accumulate charge and generate an electrostatic field, providing a passive signature independent of external radiation. This study develops a quantitative triboelectric charging model based on kinetic gas theory. A shielded high-speed airflow impact experiment estimated an equivalent charge-transfer parameter for the tested carbon-fiber composite and airflow, with an adjusted coefficient of determination (adjusted R^2^) of 0.967. Flow-field, dilute gas–solid two-phase, and multiphysics electrostatic simulations were then used to determine surface charging and near-field characteristics. Flight encounter tests employed an induced-current electrostatic detector and a MEMS electric-field sensor. At 30 m/s, the simplified model predicted a total charge on the order of 10^−7^ C and a 3.8% variation from 0° to 20° angle of attack. The maximum electric-field strength along the nominal 10 m encounter trajectory was 415 V/m. After filtering and temporal alignment, the measured and simulated electric-field rates of change had an adjusted R^2^ of 0.980 for the selected encounter, while the MEMS measurements yielded a shape-consistency coefficient of 0.937. The encounter measurements provide preliminary support for the predicted response trend under the tested conditions and a quantitative framework for analyzing the electrostatic near-field signatures and passive detection of small UAVs. The method may serve as a complementary means of short-range UAV detection.

## 1. Introduction

Small unmanned aerial vehicles (UAVs) have been widely used in inspection, surveying and mapping, environmental monitoring, reconnaissance, and security because of their low cost, small size, light weight, ease of operation, flexibility, and adaptability [1]. Current counter-UAV systems detect targets primarily by radio-frequency, electro-optical, and acoustic methods. High-resolution radar can identify the motion characteristics of small UAVs [2]. However, the small size, low flight altitude, and extensive use of composite materials result in a low signal-to-noise ratio of radar echoes, making detection stability susceptible to low-altitude clutter and complex electromagnetic environments [3]. Infrared detection can identify small UAV targets under low illumination [4], but target–background thermal contrast and complex backgrounds can limit recognition. Visible-light detection offers high recognition accuracy, but its performance depends on illumination, weather, occlusion, and background contrast and may deteriorate in adverse weather or complex backgrounds [5,6]. Acoustic detection does not depend on electromagnetic signals and offers low cost and flexible deployment [7], but it is vulnerable to strong background noise, wind disturbances, and complex propagation environments [8]. Overall, existing detection modalities retain limitations in complex environments and short-range precision-detection scenarios [9,10], particularly during close encounters, which impose stringent requirements on response speed and interference resistance.

During flight, interactions between a UAV surface and air or suspended particles may cause charge accumulation. The resulting electric field does not depend on an external radiation source and therefore has potential as a passive signature for UAV detection [11]. In terms of measurement, an induced-current electrostatic detector based on charge induction is sensitive to temporal variations in the target electric field and is suitable for near-field scenarios involving rapid target–detector encounters [12,13]. Electric-field sensors can provide an auxiliary measurement of field strength [14]. Here, passive detection means that the sensor emits no probing signal; the detector electronics still require power. However, existing studies provide insufficient modeling of the UAV triboelectric charging process, lack a systematic method linking flow-field characteristics to electric-field characteristics, and offer limited comparisons between simulations and experiments under practical detection scenarios.

Aircraft charging has been studied experimentally. Jensen [15] reported field-meter measurements using drone and helicopter setups and confirmed helicopter charging in the available flight record. Gas–solid contact electrification has also been investigated [16,17], including experiments using gas jets directed at metal electrodes [17]. These studies support the investigation of airflow-related charging, while the contribution of each charge source depends on the material and test conditions.

To address these issues, this study investigates triboelectric charging during UAV flight. A UAV triboelectric charging model is established from kinetic gas theory, and its key equivalent parameter is estimated experimentally for one airflow and material. Flow-field simulations of a simplified model of a representative UAV are then used to calculate the surface-charge distribution and analyze triboelectric charging behavior, followed by simulations of the electric-field characteristics and electrostatic detection scenario. Finally, flight tests are performed to compare simulated and measured response trends under a specified operating condition.

## 2. Triboelectric Charging Mechanism of a Low-Altitude UAV

### 2.1. Development of the Triboelectric Charging Model

For small UAVs, interaction between the surface and surrounding air may contribute to charge accumulation. Studies of other gas–solid systems suggest that electron transfer is a possible mechanism [16,17]. Ions, particles, droplets, and material transfer may also contribute under the present airflow conditions. Initially neutral particles can exchange charge upon contact with the surface, while charged particles can also carry charge to it. Water vapor can affect surface properties, and condensed droplets can contribute through liquid–solid contact. For an equivalent approximation, the mean molecular mass of air is adopted; the calculation does not distinguish nitrogen, oxygen, and water vapor. Because charge transfer at a material–gas interface is complex, the internal structures of the gas molecules and modes of molecular motion other than translation are generally neglected when describing molecular collisions [18], as are van der Waals and dispersion forces during collisions between the surface material and gas molecules [19].

According to kinetic gas theory, when many gas molecules collide frequently with a solid surface, the macroscopic effect of those collisions is the pressure exerted by the gas on that surface [20]. Therefore, when an object moves through air, surface pressure can be used to estimate an equivalent interaction rate; pressure alone does not show which encounters transfer charge.

Consider a surface element *dA* whose normal is aligned with the incident flow within the effective collision region of the UAV. Let its velocity relative to the airflow be *v_G_*. After a flight time *dt*, the element sweeps a cylindrical volume with base area *dA* and height *v_G_dt*, and this volume is used to estimate the number of possible encounters. This construction provides an encounter estimate for normal incidence; it does not directly describe tangential flow over a wing or fuselage. Let the number of molecules in this volume be *dN_G_*, and let the molecular number density of air be *n_c_* (molecules/m^3^). Because the UAV operates at a low Mach number, the surrounding gas is not strongly compressed, and its temperature does not change markedly; thus, the ideal-gas equation remains applicable [20]. The molecular number density is therefore obtained as follows [21]:

(1) $$\left\{\begin{array}{c} n_{c}=\frac{N_{A}n}{V}\\ \frac{n}{V}=\frac{P_{S}}{RT} \end{array}\right.,$$

(2) $$n_{c}=\frac{N_{A}P_{s}}{RT},$$

where *N_A_* is the Avogadro constant (6.02 × 10^23^ molecules/mol), *n* is the amount of substance (mol), *V* is the gas volume (m^3^), *P_s_* is the static pressure (Pa), *R* is the molar gas constant (8.314472 J/(mol·K)), and *T* is the ambient temperature (K).

After a flight time *dt*, the estimated number of molecular encounters for a surface facing the flow is

(3) $$dN_{G}=v_{G}dAdtn_{c}=\frac{N_{A}P_{s}}{RT}v_{G}dAdt,$$

The mean molecular mass of air is approximated as *m*_0_ = 4.82 × 10^−26^ kg. The total mass *dm* of gas molecules colliding with the surface element during *dt* is *m*_0_*dN_G_*, therefore,

(4) $$2m_{0}dN_{G}v_{G}=P_{d}dAdt,$$

where *P_d_* is the dynamic pressure (Pa) [22]. The corresponding number of gas molecules is

(5) $$dN_{G}=\frac{P_{d}dAdt}{2m_{0}v_{G}},$$

Equations (3) and (5) express the collision count using static-pressure and dynamic-pressure relations, respectively. Solving the two equations simultaneously and eliminating *v_G_* gives an equivalent encounter count for the assumed flow geometry during *dt*:

(6) $$dN_{G}=\sqrt{\frac{N_{A}P_{s}P_{d}}{2m_{0}RT}}dAdt,$$

The parameter *k*(*t*) represents the measured net charge increment divided by the equivalent encounter count in the airflow experiment. It is an empirical parameter for this material and test condition, rather than the charge transferred in one gas-molecule collision. Its value may also reflect charge loss during exposure.

Within the normal-incidence approximation used to derive the equivalent encounter rate, the estimated charge increment on a surface element over *dt* is given by Equation (7):

(7) $$dQ_{G}=k(t)\sqrt{\frac{N_{A}P_{s}P_{d}}{2\bar{m}RT}}dAdt,$$

After a period *τ*, the surface charge density is

(8) $$\sigma_{G}(\vec{r})=\int_{\tau }k(t)\sqrt{\frac{N_{A}P_{s}P_{d}}{2m_{0}RT}}dt,$$

The equivalent charging-current density on the UAV surface is

(9) $$J_{G}(\vec{r})=\frac{d\sigma_{G}(\vec{r})}{dt}=k(t)\sqrt{\frac{N_{A}P_{s}P_{d}}{2m_{0}RT}},$$

Integrating the current density over the UAV surface gives the total triboelectric charging current:

(10) $$I_{G}=\oint_{s}J_{G}ds=\oint_{s}k(t)\sqrt{\frac{N_{A}P_{s}P_{d}}{2m_{0}RT}}ds,$$

After a flight duration *τ*, the charge estimated by the equivalent model for the UAV surface is

(11) $$Q_{G}=\int_{\tau }\oint_{s}k(t)\sqrt{\frac{N_{A}P_{s}P_{d}}{2m_{0}RT}}dsdt,$$

In the equivalent model, the estimated charge depends on the surface pressures, the effective interaction area, and the parameter calibrated for the tested material and airflow. The measured parameter describes net charge change during airflow exposure; it does not separately identify charge generation and charge loss. The retained charge may also depend on surface resistivity, electrical connections, humidity, and exposure time. Accordingly, predictions for other materials or airflow geometries require separate calibration or validation.

The model represents the complex electrification process at the gas–solid interface by the collision frequency and the condition-dependent parameter *k*(*t*) [23]. In Section 2.2, the equivalent parameter of a carbon-fiber composite is calibrated using standard synthetic air at a relative velocity of 30 m/s under the specified laboratory temperature and humidity. It should therefore not be interpreted as a universal material constant independent of the experimental conditions. For low-altitude environments containing small amounts of dust, sand, and other solid particles, the particle-laden air can subsequently be modeled as a gas–solid two-phase flow. In the present simulation, the particles affect the flow-field parameters and hence the model estimate; their direct electrical contribution is not calculated.

The model thus shows that the UAV surface-charge characteristics can be quantified by obtaining the surface-pressure distribution through flow-field simulation and combining it with the triboelectric charging model. This provides the basis for the subsequent analyses of electric-field characteristics and electrostatic detection.

### 2.2. Determination of the Key Parameter

Quantitative evaluation of triboelectric charge requires the key model parameter—the equivalent charge-transfer parameter—to be determined. An experiment was therefore conducted to measure triboelectric charging during interaction between a carbon-fiber composite and an airflow. Because small-UAV surfaces are commonly made of carbon-fiber composites and their typical speed range is 20–40 m/s, the charge response of a carbon-fiber composite plate was measured under a 30 m/s airflow. The charge-transfer relation under the specified experimental conditions was then fitted from these measurements.

According to kinetic gas theory, the number of collisions between gas molecules and a unit area of the carbon-fiber plate per unit time is

(12) $$Z(\vec{r},t)=\frac{P(\vec{r},t)}{\sqrt{2\pi \bar{m}k_{B}T}},$$

where *P* is the gas pressure on the carbon-fiber plate surface (Pa), *k_B_* is the Boltzmann constant, and *T* is the gas temperature (K).

The total number of collisions between gas molecules and the carbon-fiber plate per unit time is therefore

(13) $$Z(t)=\iint_{A}\frac{P(\vec{r},t)}{\sqrt{2\pi \bar{m}k_{B}T}}dA,$$

where *A* is the surface area of the carbon-fiber plate (m^2^).

Here, $\iint_{A}P(\vec{r},t)dA$ is the force exerted on the carbon-fiber plate by the impinging airflow. Because a pressure sensor would alter the direction and magnitude of the airflow interacting with the plate, this force could not be measured directly. Instead, a precision balance was used to measure the apparent weight increment caused by the airflow in real time, from which the impact force on the plate was calculated indirectly. Equation (13) can therefore be rewritten as

(14) $$Z(t)=\frac{\Delta m(t)g}{\sqrt{2\pi \bar{m}k_{B}T}},$$

A Faraday cup connected to an electrometer was used to measure the temporal variation in the charge carried by the carbon-fiber plate during airflow exposure and hence the charge increment on the plate as a function of time.

Under the equivalent model, the measured charge increment per unit time was temporally aligned with the equivalent encounter-count sequence, and their ratio yielded the equivalent charge-transfer parameter for the tested carbon-fiber composite and airflow under the experimental condition:

(15) $$k_{30,C}(t)=\frac{\Delta q(t)}{Z(t)}=\frac{\Delta q(t)\sqrt{2\pi \bar{m}k_{B}T}}{\Delta m(t)g},$$

**Figure 1:**
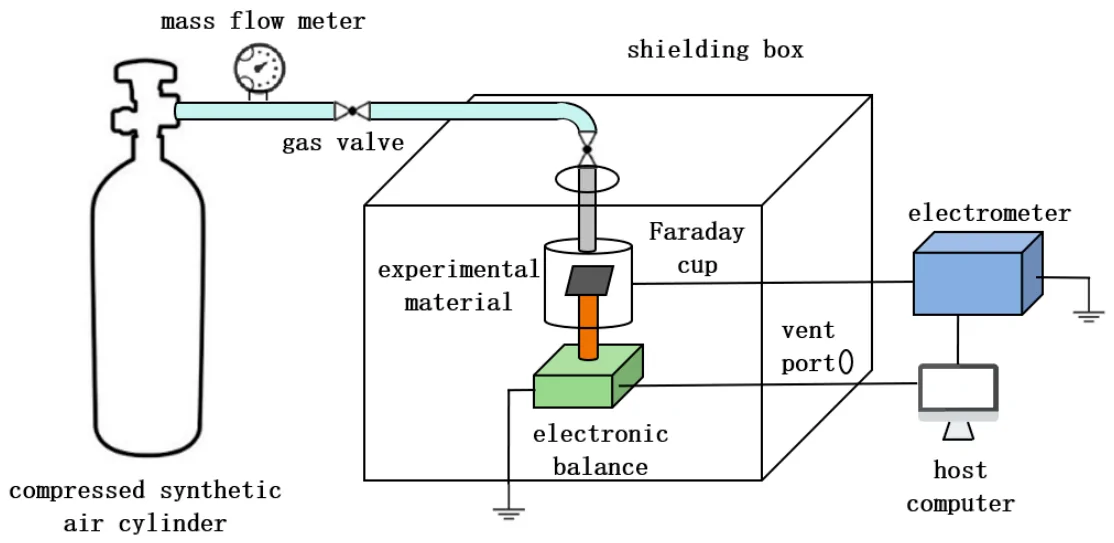
Experimental system schematic.

The experimental system shown in Figure 1 was designed and constructed. To provide a controlled air supply for the charging experiment, a 40 L cylinder containing 99% pure synthetic air at a working pressure of 15 MPa was used with a two-stage pressure-reducing valve and a pressure gauge for real-time monitoring. The gas line comprised PVC and stainless-steel tubing with an inner diameter of 8 mm. Owing to the cylinder-outlet configuration and site constraints, flexible PVC tubing was connected to the cylinder, whereas grounded stainless-steel tubing was used inside the shielded enclosure to reduce charge carried by the airflow. Two rotary valves controlled the gas flow and connected the tubing sections. A flowmeter with a range of 10–100 L/min measured the supply flow rate, which was converted to gas velocity from the tube diameter. Experiments were performed in an acrylic glove-type shielded enclosure lined internally with 200-mesh copper screen to isolate external electric fields and stray charge. A 0.1 m diameter opening in the enclosure roof accommodated the clamped stainless-steel tube, and several exhaust openings prevented overpressure during rapid discharge. Carbon-fiber composite coupons measuring 40 mm × 40 mm (A = 0.0016 m^2^) were used. Their side length exceeded the gas-line diameter. The stainless-steel outlet was centered above the Faraday cup and aligned normal to the coupon surface before each experiment. The coupon was connected to the balance by a small-diameter, lightweight aluminum tube that provided rigid support, and it was positioned at the center of the Faraday cup. To prevent charge generated by airflow interaction with the aluminum tube and balance from affecting the measurement, the tube and the balance pan were wrapped in copper-foil tape and grounded. Insulating tape between the coupon and tube reduced dissipation of accumulated coupon charge. Before each experiment, the coupon surface was cleaned with anhydrous ethanol to remove contaminants and residual charge, and the balance was tared. Charge was measured without direct electrical contact by connecting the Faraday cup to an electrometer and recording the charge variation in real time. The connection cable was covered with metallic mesh to shield against unrelated electric-field interference. The principal instrument specifications are listed in Table 1. The calibrated *k*(*t*) characterizes the net charging response of the tested jet–specimen system. The supplied gas composition and laboratory humidity are documented above and in Table 1. Aerosol concentration, incoming charge, and humidity at the specimen were not measured separately, and the airflow was used without additional filtering or neutralization. These conditions allow *k*(*t*) to describe the combined charging response of the airflow and specimen, with the individual charge sources remaining indistinguishable.

The charge and airflow-impact force were measured as functions of time, and the equivalent charge-transfer parameter was inferred using Equation (15). Fitting the measurements yielded the following parameter function for the carbon-fiber composite under a 30 m/s airflow:

(16) $$k_{30,C}(t)=7.59\times 10^{-34}e^{-0.19t}-5.02\times 10^{-34}e^{-0.18t},$$

**Figure 2:**
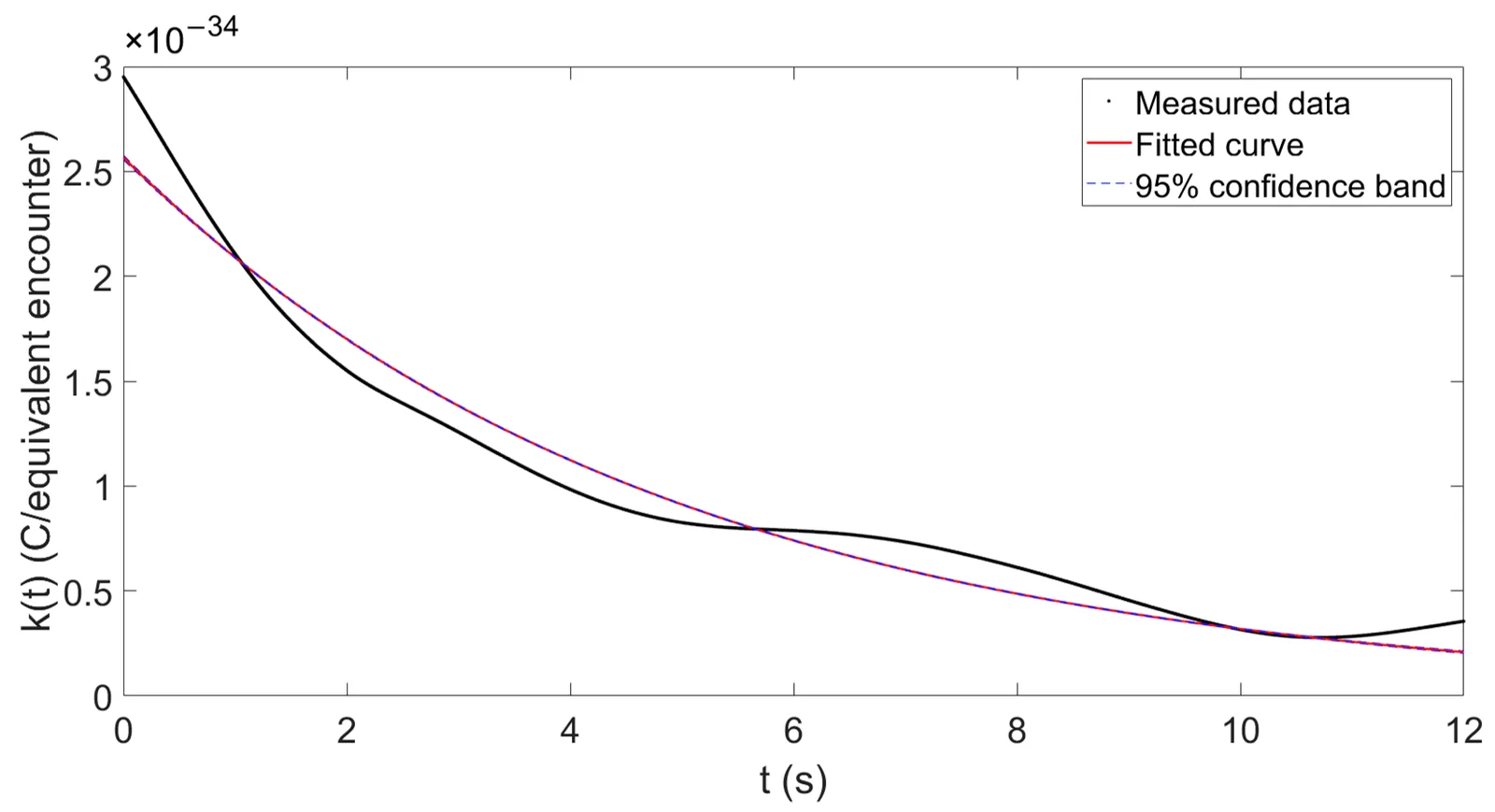
Equivalent charge-transfer parameter *k*(*t*) of the carbon-fiber composite under a 30 m/s airflow. The denominator represents the equivalent encounter count defined in the model. Black: measured values; red: fitted curve; blue dashed: 95% confidence band.

The fit yielded an adjusted coefficient of determination (adjusted R^2^) of 0.967; the 95% confidence band is shown in Figure 2. This fit characterizes the tested carbon-fiber composite under a 30 m/s airflow.

As shown in Figure 2, under the present experimental conditions and equivalent model, *k*(*t*) of the carbon-fiber composite decreases gradually toward zero, consistent with a gradual slowing of net charge accumulation as charge generation and dissipation approach a balance under the tested airflow conditions.

Substituting Equation (16) into Equations (7)–(11) yields the surface charge density, triboelectric charging current, and accumulated charge of a carbon-fiber UAV at a freestream velocity of 30 m/s.

In the calibration experiment, the carbon-fiber plate was stationary and exposed to a 30 m/s airflow, whereas in actual flight the UAV moves relative to the surrounding gas. The calibration jet strikes the plate normally, whereas the flow over much of the flying UAV surface is tangential. Their boundary layers and contact conditions differ even at the same nominal relative speed. Accordingly, the calibrated *k*(*t*) provides a first estimate for the same surface material near 30 m/s in the flight simulation. Gas-jet experiments have been used to investigate gas–solid charge transfer [17], supporting their use for studying charging under controlled conditions. Here, the simulated surface pressures account for changes in the macroscopic flow, while the use of the jet-derived *k*(*t*) is a first-order approximation. A quantitative correction for the different flow geometries would require calibration under tangential-flow conditions.

## 3. Multiphysics Simulation of UAV Electrostatic Characteristics and Detection

### 3.1. UAV–Air Triboelectric Charging Characteristics

#### 3.1.1. UAV Geometry

**Figure 3:**
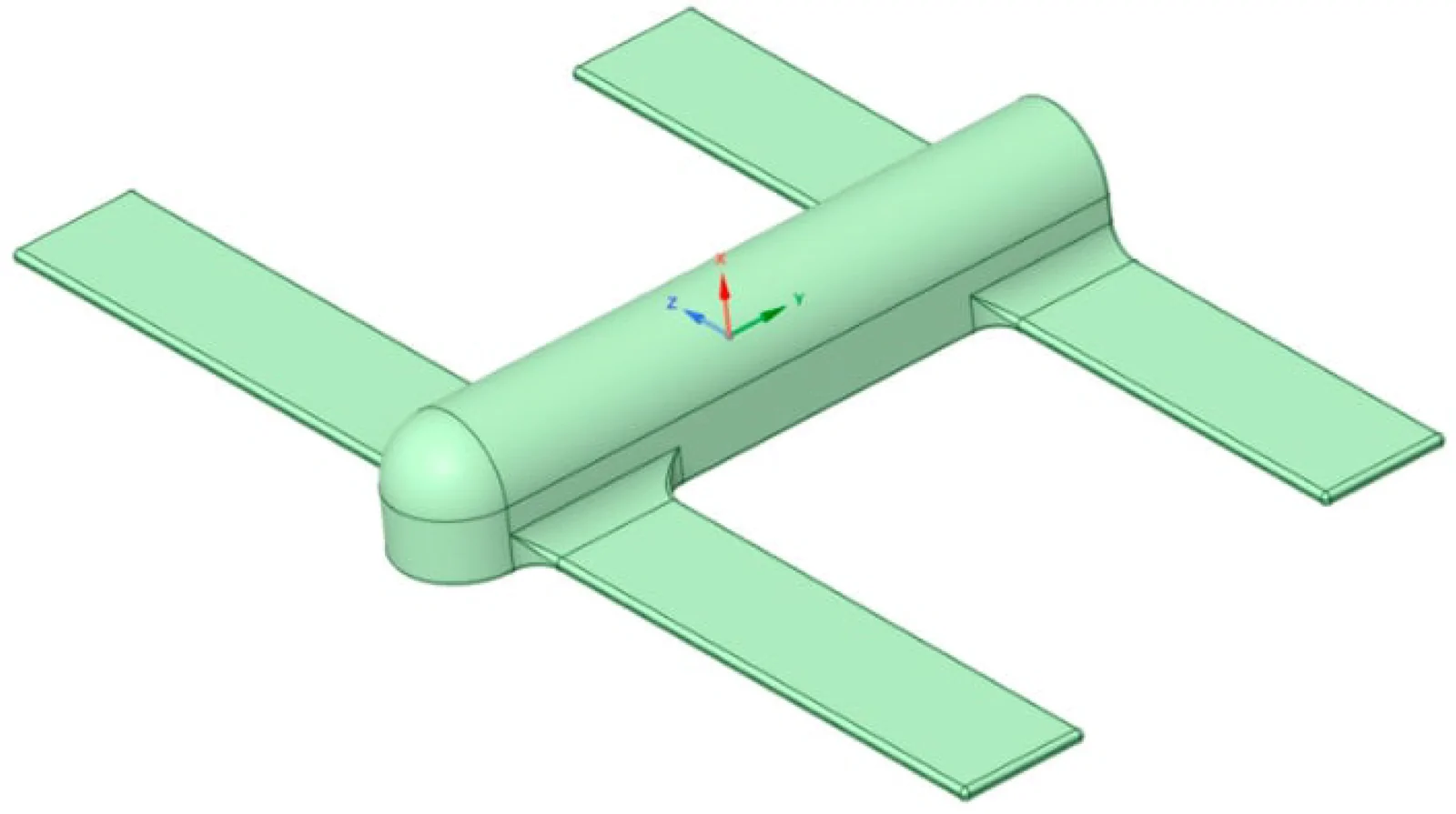
Three-dimensional model of the UAV.

A representative small fixed-wing UAV was selected, and a simplified three-dimensional geometry that retained its principal aerodynamic features was developed for numerical simulation. The UAV center of mass was defined as the coordinate origin, and the roll axis (the longitudinal axis passing through the center of mass and parallel to the flight direction) was aligned with the y-axis of the simulation software. The model dimensions are listed in Table 2, and the three-dimensional model is shown in Figure 3.

**Figure 4:**
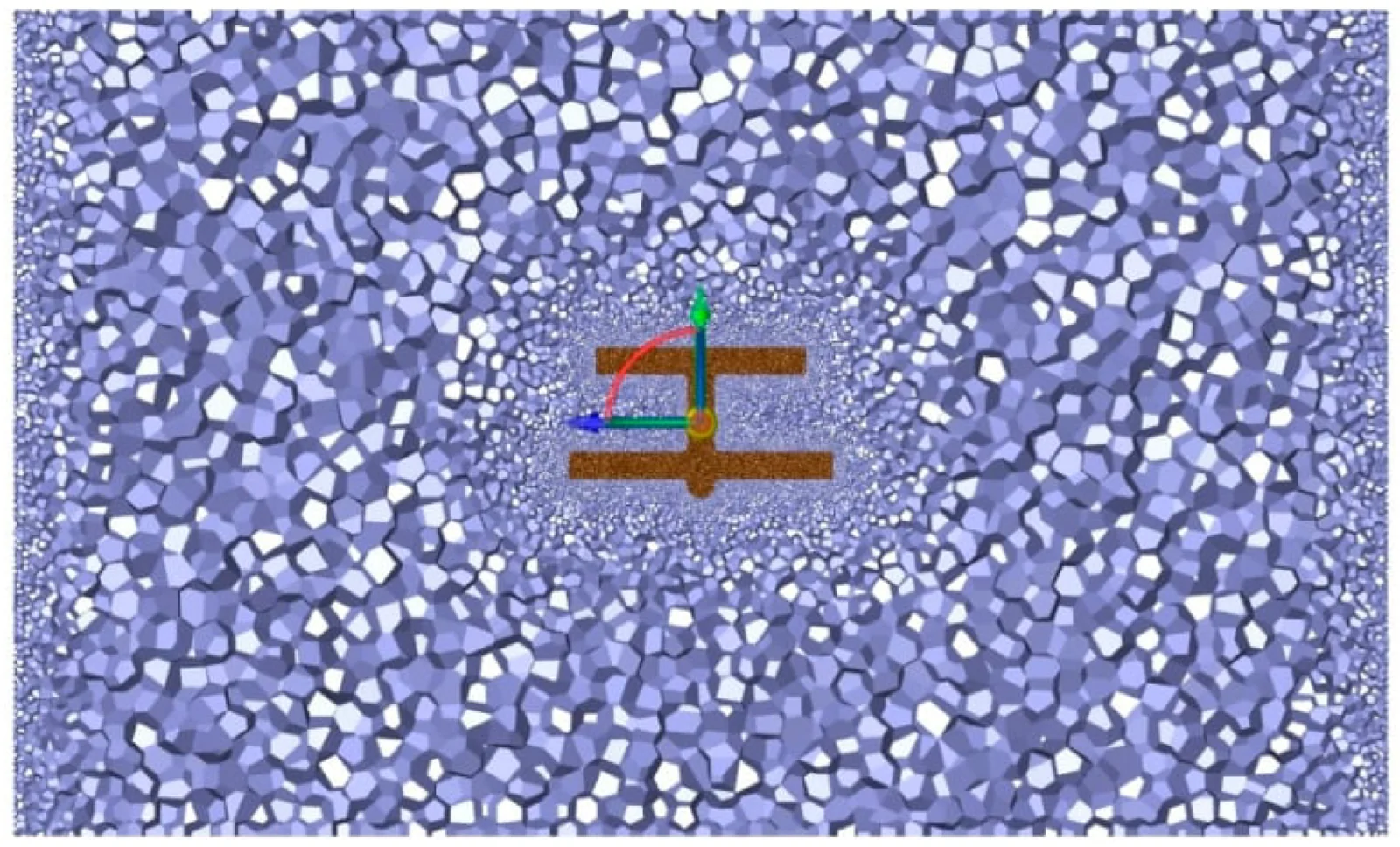
Mesh of the UAV model. The purple cells represent the computational domain, with local mesh refinement around the UAV shown in brown. The straight arrows indicate the model coordinate axes, with the origin at the UAV center of mass and the longitudinal roll axis aligned with the y-axis. The curved arrow indicates the orientation between the displayed coordinate axes.

The computational domain was meshed with local refinement in critical regions. The final mesh comprised 1,442,075 cells, 9,690,048 faces, and 7,862,603 nodes, as shown in Figure 4.

#### 3.1.2. Simulation of Surface Triboelectric Charging

The freestream velocity was set to 30 m/s, the angle of attack to 0°, the flight altitude to 80 m, the temperature to 288 K, and the operating pressure was set to 100,913 Pa. The realizable k–ε two-equation turbulence model with enhanced wall treatment was adopted, and viscous dissipation was included. The convergence criteria were residuals below 10^−4^ for continuity, 10^−3^ for momentum, and 10^−6^ for energy. After initialization, all residuals fell below the specified thresholds after 182 iterations, satisfying the convergence criteria used in this study.

**Figure 5:**
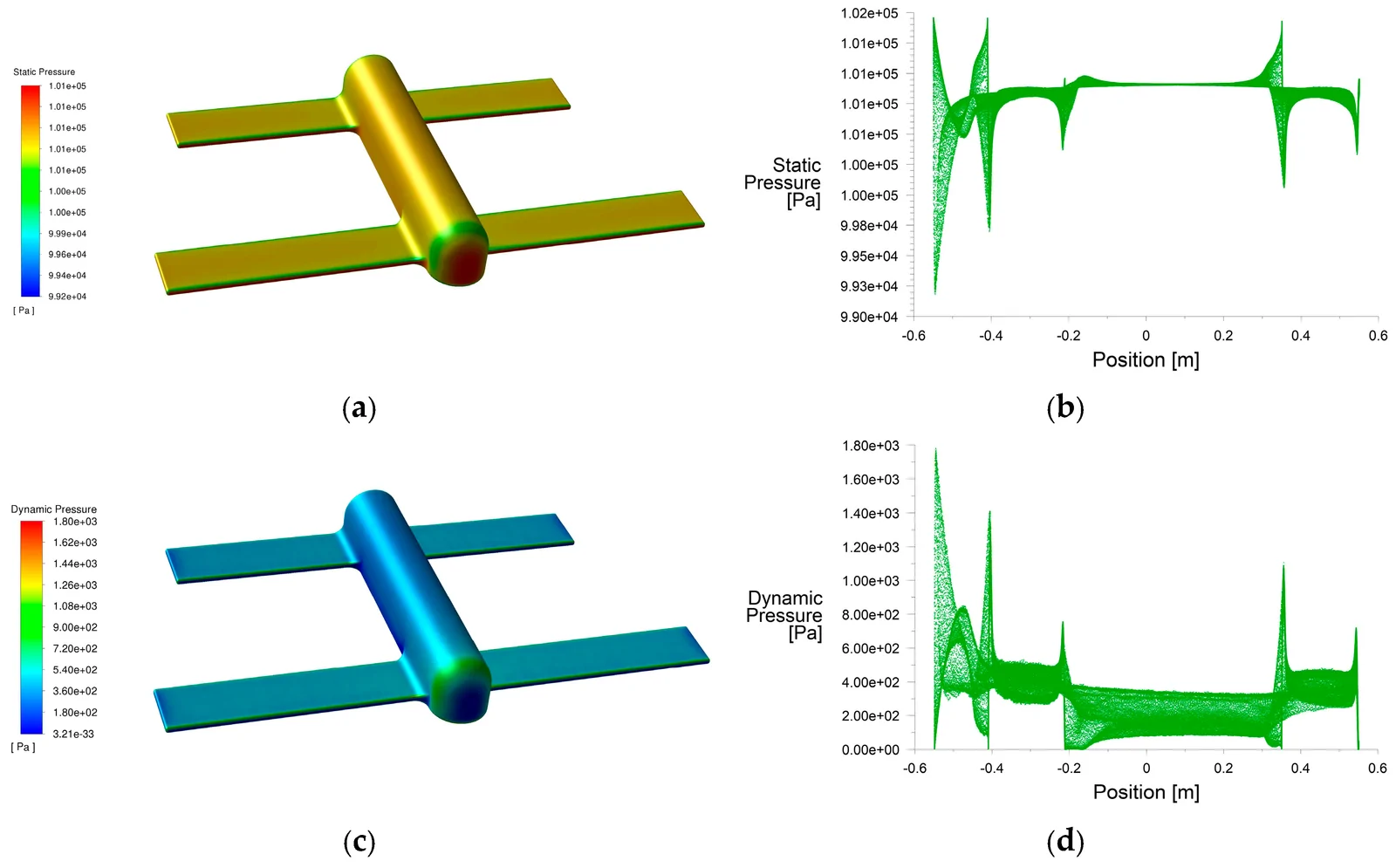
Static- and dynamic-pressure distributions on the UAV surface at an angle of attack of 0°: (**a**) Surface static-pressure contour. (**b**) Surface static-pressure range. (**c**) Surface dynamic-pressure contour. (**d**) Surface dynamic-pressure range.

The simulated static and dynamic pressures on the UAV surface are shown in Figure 5.

As shown in Figure 5, at an angle of attack of 0°, the surface pressure varies primarily along the freestream direction. The foremost fuselage surface and wing leading edges exhibit high static pressure and low dynamic pressure, whereas rounded regions exhibit low static pressure and high dynamic pressure. Pressure is relatively uniform along the freestream direction over the middle fuselage, while the tail edges exhibit low static pressure and high dynamic pressure. The maximum and minimum static pressures are 1.014 × 10^5^ and 9.918 × 10^4^ Pa, respectively, and the maximum and minimum dynamic pressures are 1781.36 and 0.137 Pa, respectively.

**Figure 6:**
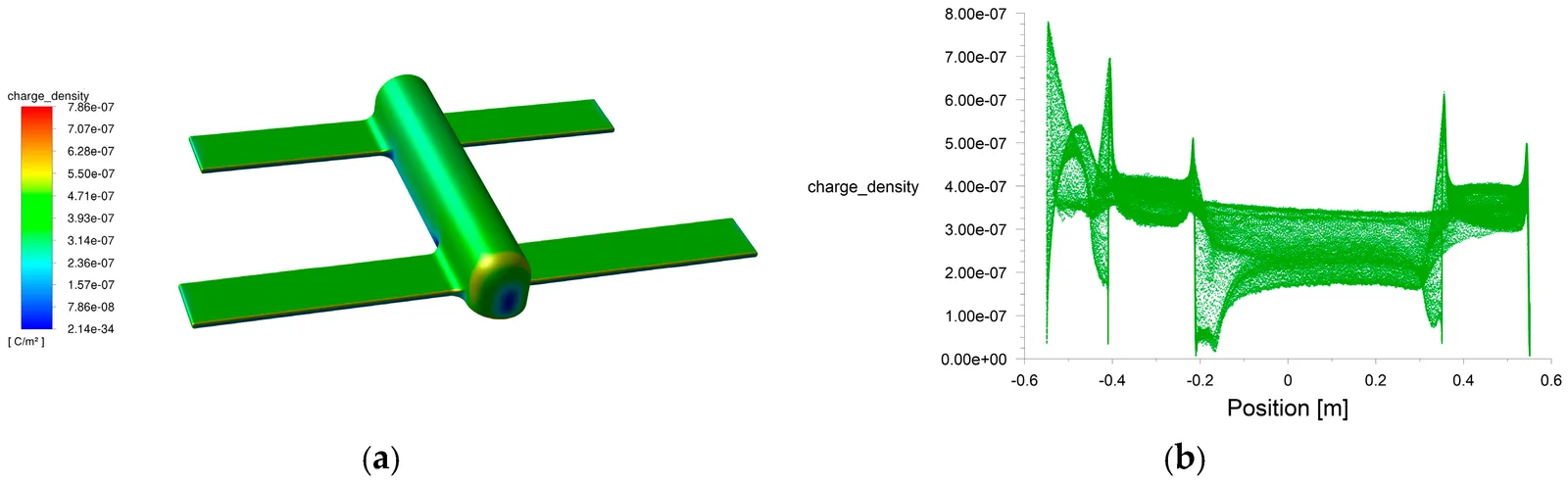
(**a**) UAV surface charge density at an angle of attack of 0°. (**b**) UAV surface charge density range at an angle of attack of 0°.

According to Equation (8), the surface charge density can be obtained from the charge-transfer parameter and the surface static and dynamic pressures. Equation (8), together with Equation (16), was implemented using named expressions in ANSYS Fluent 2021 R1 Fluent to calculate the surface charge density of the carbon-fiber UAV at a freestream velocity of 30 m/s, as shown in Figure 6.

As shown in Figure 6, at an angle of attack of 0°, the surface charge density is low at the foremost fuselage surface and wing leading edges. It is higher around the rounded fuselage and wing regions and at the tail edges. Over the middle fuselage, which is parallel to the freestream direction, the charge density is low and comparatively uniform. The maximum and minimum surface charge densities are 7.804 × 10^−7^ and 6.790 × 10^−9^ C/m^2^, respectively.

Area integration of the UAV surface charge density gives a total triboelectric charge of approximately 7.652 × 10^−7^ C for the simplified model at an angle of attack of 0°.

#### 3.1.3. Triboelectric Charging at Different Angles of Attack

Changing the angle of attack alters the relative motion between each UAV surface element and the airflow, thereby changing the surface static- and dynamic-pressure distributions and, consequently, the surface charge density and total triboelectric charge. To assess this effect under representative flight conditions, surface charge density and total charge were simulated at angles of attack of 10°, 15°, and 20°. The results are listed in Table 3.

As shown in Table 3, as the angle of attack increases from 0° to 20°, the maximum surface charge density decreases from 7.804 × 10^−7^ to 7.433 × 10^−7^ C/m^2^, and the total triboelectric charge decreases from 7.652 × 10^−7^ to 7.363 × 10^−7^ C. The relative change is approximately 3.8%, while the order of magnitude remains unchanged. Thus, within the simplified model and angle-of-attack range considered here, the angle of attack has a relatively limited effect on the integrated charge.

### 3.2. Overall Triboelectric Charging Characteristics of the UAV

Because small UAVs generally operate at low altitudes, they interact not only with air but also with airborne dust, sand, and other particles during flight [24]. The influence of interactions between the UAV and such particles should therefore be considered when evaluating its overall triboelectric charging.

Under typical UAV flight conditions, dust and sand particles have low concentrations, small diameters, and small volume fractions, and the interaction between the particle and gas phases is weak. The flow can therefore be treated as a dilute two-phase system. Coupling the airflow and particle flow in the simulation enables the pressure-based charge estimate to be compared under representative environmental conditions.

A discrete phase model (DPM) was added to the original flow simulation. The particle velocity was set equal to the freestream velocity of 30 m/s, and the angle of attack was 0°. Based on particle data for representative terrestrial terrain and weather conditions [25,26], particles following a Rosin–Rammler distribution were injected from the inlet. The total particle mass flow rate was 4 × 10^−5^ kg/s; the minimum, maximum, and mean particle diameters were 4 × 10^−6^, 3 × 10^−5^, and 1 × 10^−5^ m, respectively; the spread parameter was 3; and the particle temperature was 288 K. Because the density of dust particles depends primarily on their mineral composition and particles larger than 1 μm generally consist of quartz and aluminosilicates, the particle density was set to 2650 kg/m^3^.

**Figure 7:**
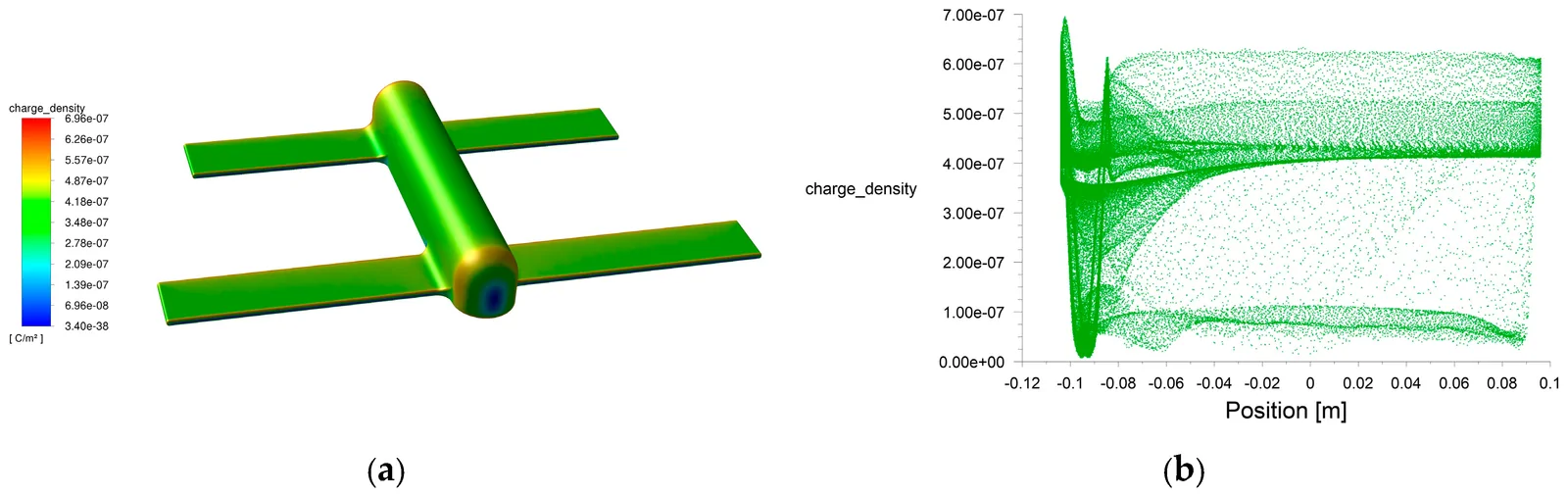
Flow-field-based estimate of UAV surface charge density under the specified dilute-particle condition: (**a**) UAV surface charge density. (**b**) UAV surface charge density range.

After inclusion of the particle phase, the surface charge density estimated from the modified surface flow-field parameters using the same equivalent gas–material model is shown in Figure 7. Its maximum and minimum values were 6.95 × 10^−7^ and 8.10 × 10^−9^ C/m^2^, respectively. Under the specified dilute-particle condition, the estimated total surface charge increased by 11.5% relative to the air-only calculation. This appreciable difference shows the influence of the selected particle phase on the flow-field-based charge estimate. The corresponding surface-charge distribution retained a broadly similar pattern.

### 3.3. UAV Electric-Field Characteristics

The electric field around a UAV in flight depends on the charge accumulated over its surface. Once the surface charge density has been obtained, the electric-field strength and electric-potential distribution in the surrounding space can be calculated. For this purpose, the UAV surface charge-density data at an angle of attack of 0° calculated in ANSYS Fluent were imported into COMSOL Multiphysics 6.0 Multiphysics to establish the electrostatic simulation model.

**Figure 8:**
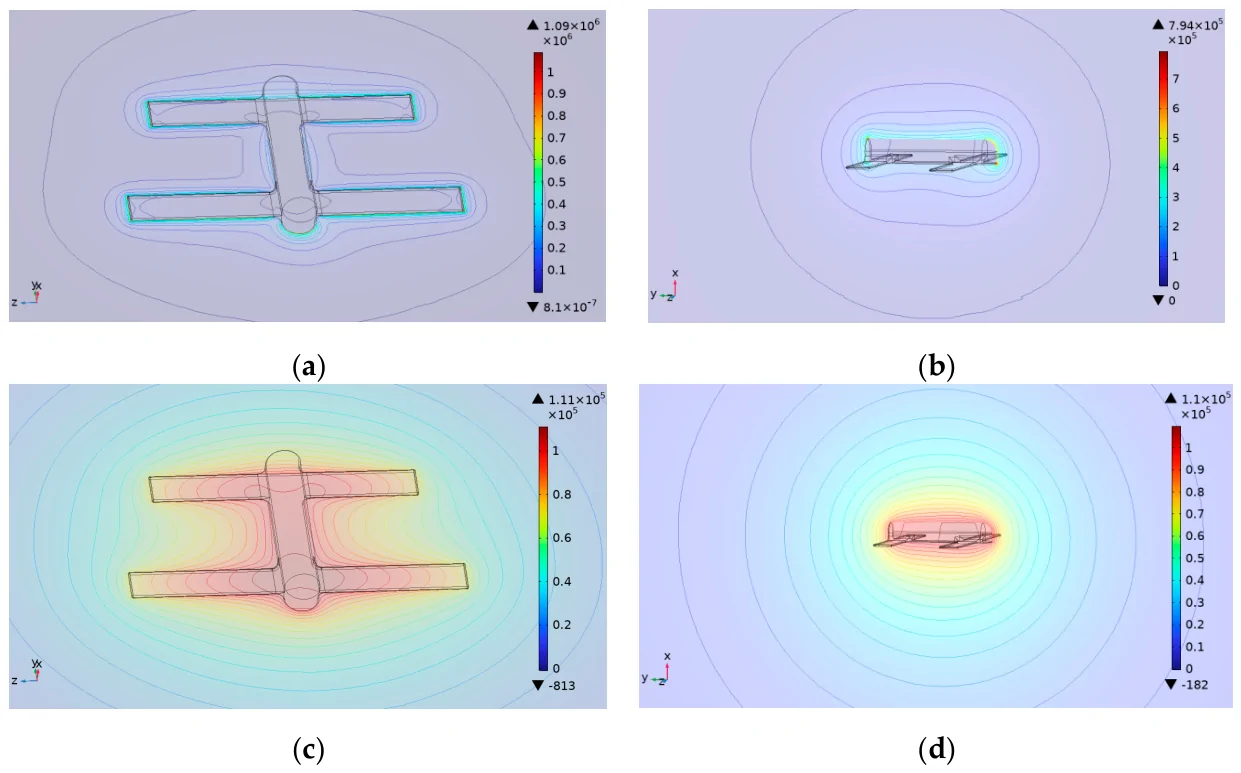
Electric-field and electric-potential contours around the UAV: (**a**) Electric-field contour on the YOZ plane. (**b**) Electric-field contour on the XOY plane. (**c**) Electric-potential contour on the YOZ plane. (**d**) Electric-potential contour on the XOY plane.

The simulated electric-field and electric-potential distributions around the UAV are shown in Figure 8.

As shown in Figure 8, the electric-field and electric-potential distributions around the UAV are closely related to its surface-charge distribution. The electric-field strength is highest near the vehicle surface and decays rapidly with distance. Local field-enhancement regions occur adjacent to areas of high surface charge density, including the rounded fuselage and wing regions and the tail edges. These results show that a UAV in flight can form an electric field with a distinct spatial distribution, providing a physical basis for electrostatic detection.

### 3.4. Simulation of UAV Electrostatic Detection

To evaluate the model in a target-detection scenario, the encounter between a carbon-fiber composite UAV and an electrostatic detection system was simulated. Consistent with the flight-test geometry, a three-dimensional cut line parallel to the UAV roll axis and located 10 m above the simplified UAV model was defined as the encounter trajectory. The sensing-electrode plane was parallel to the UAV roll and pitch axes. Accordingly, the field quantity relevant to electrostatic induction was the component normal to this plane, E_n_ = E · n, where n is the unit normal to the electrode plane.

**Figure 9:**
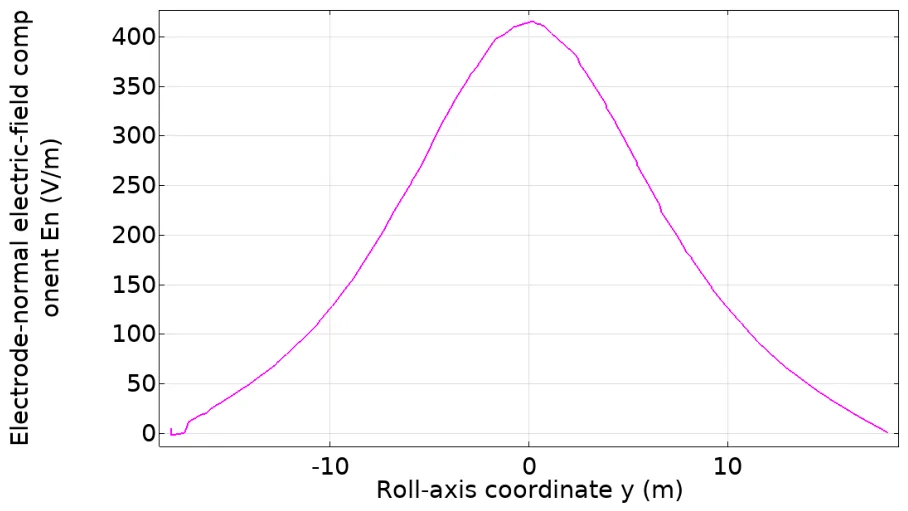
Normal component of the electric field, E_n_ = E · n, along the encounter trajectory 10 m above the UAV as a function of the roll-axis coordinate y.

As shown in Figure 9, the normal electric-field component increased as the horizontal separation from the UAV decreased. Its maximum along the specified trajectory was 415 V/m.

**Figure 10:**
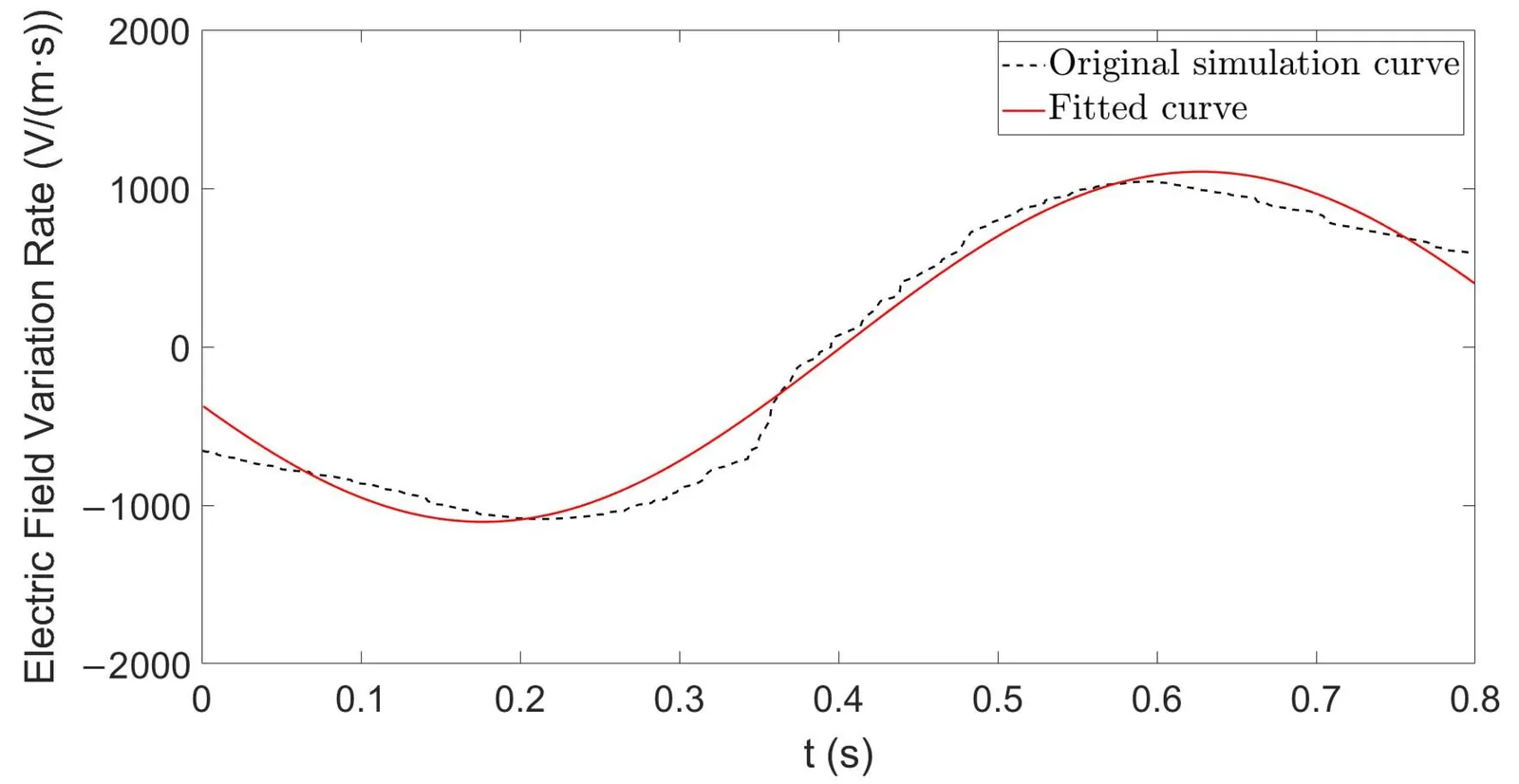
Simulated rate of change of the electrode-normal electric-field component, dE_n_/dt, along the encounter trajectory 10 m above the UAV. Black dashed line: simulated data; red line: fitted curve.

The simulated rate of change dE_n_/dt along the 10 m encounter trajectory was then calculated using the relative motion of the UAV and detector. Because numerical differentiation can amplify local fluctuations, the simulated response was smoothed and fitted. The selected fitting function yielded an adjusted coefficient of determination of 0.981 (Figure 10). The resulting temporal signature was compared with the rate inferred from the induced current in Section 4.

These results indicate that the electric-field rate of change has a distinct temporal signature along the specified encounter trajectory. The following section compares this signature with the response extracted from the flight-test measurements.

## 4. Experimental Validation of Electrostatic Detection

### 4.1. Electrostatic Near-Field Detection Method

A moving charged target can be detected by measuring physical quantities such as its electric-field strength, electric-field rate of change, or potential difference. This study uses an induced-current electrostatic detection method. When the detector and target approach each other rapidly, the target field at the detector changes quickly because of their changing relative position. The induced charge on the sensing electrode consequently varies, producing an induced current through the sampling resistor and an output signal in the passive detector [11]:

(17) $$I(t)=\varepsilon_{0}(1+K)\int_{S}\frac{dE_{n}(t)}{dt}dS$$

As indicated by Equation (17), measurement of the induced current provides information about changes in the local electric field [13]. Here, *ε_0_* is the permittivity of free space, *E_n_* is the component of the local electric field normal to the electrode, and *S* is the sensing-electrode area. The geometric correction in Equation (17), *K*, is a nonlinear function determined by the shape, volume, and other geometric parameters of the electrode; a geometric correction coefficient of *K* = 0.02 was used in this study. Because the sensing electrode is small relative to the target and has a regular, planar shape, the electric-field rate of change can be considered uniform over the electrode. The induced current is therefore proportional to the electric-field rate of change.

**Figure 11:**
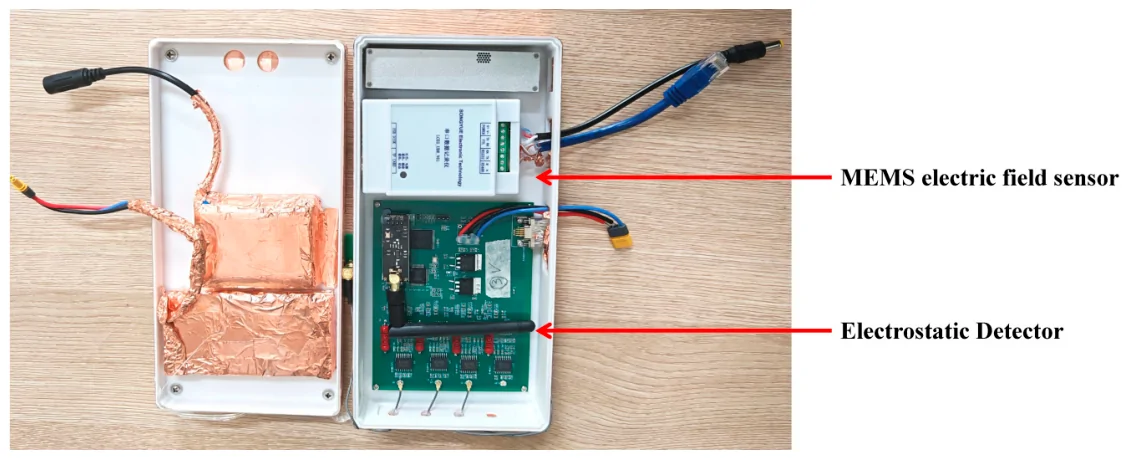
Electrostatic detection system.

The electrostatic detection system shown in Figure 11 was designed and assembled. An induced-current electrostatic detector measured the current signal during the encounter, while a MEMS electric-field sensor provided an auxiliary record of the electric-field variation. The electrostatic detector comprised a sensing electrode, preamplifier, low-pass filter, and data-acquisition unit. The preamplifier used an INA116 instrumentation amplifier, with a differential input impedance greater than 10^15^ Ω in parallel with 0.2 pF, a common-mode input impedance greater than 10^15^ Ω in parallel with 7 pF, and a typical input bias current of 3 fA at 25 °C, making it suitable for high-impedance measurement of weak electrostatic induction signals. The INA116 gain resistor RG was 20 kΩ, corresponding to a gain of 3.5, and the sampling resistor was 10 GΩ. The system was DC-coupled. The low-pass filter used R = 15 kΩ and C = 470 nF, corresponding to a characteristic frequency of approximately 22.6 Hz. Data were acquired using a 16-bit AD7606 with a ±10 V input range, a 2.5 V reference, and a sampling rate of 1 kHz; timing was controlled by an STM32F407 microcontroller, and the system supply was ±9 V. The sensing-electrode area S was 0.0023 m^2^. The auxiliary MEMS electric-field sensor was a JDC-W03A (Zhongke Feilong Sensing Technology Co., Ltd., Beijing, China), with a range of ±100 kV/m, a resolution of 5 V/m, an accuracy better than 5%, and a total parallel-plate calibration uncertainty of 2.98%. Except for the sensing electrode, the exterior of the device was shielded with copper-foil tape to reduce interference from external electric fields.

### 4.2. UAV Electrostatic Detection Experiment

**Figure 12:**
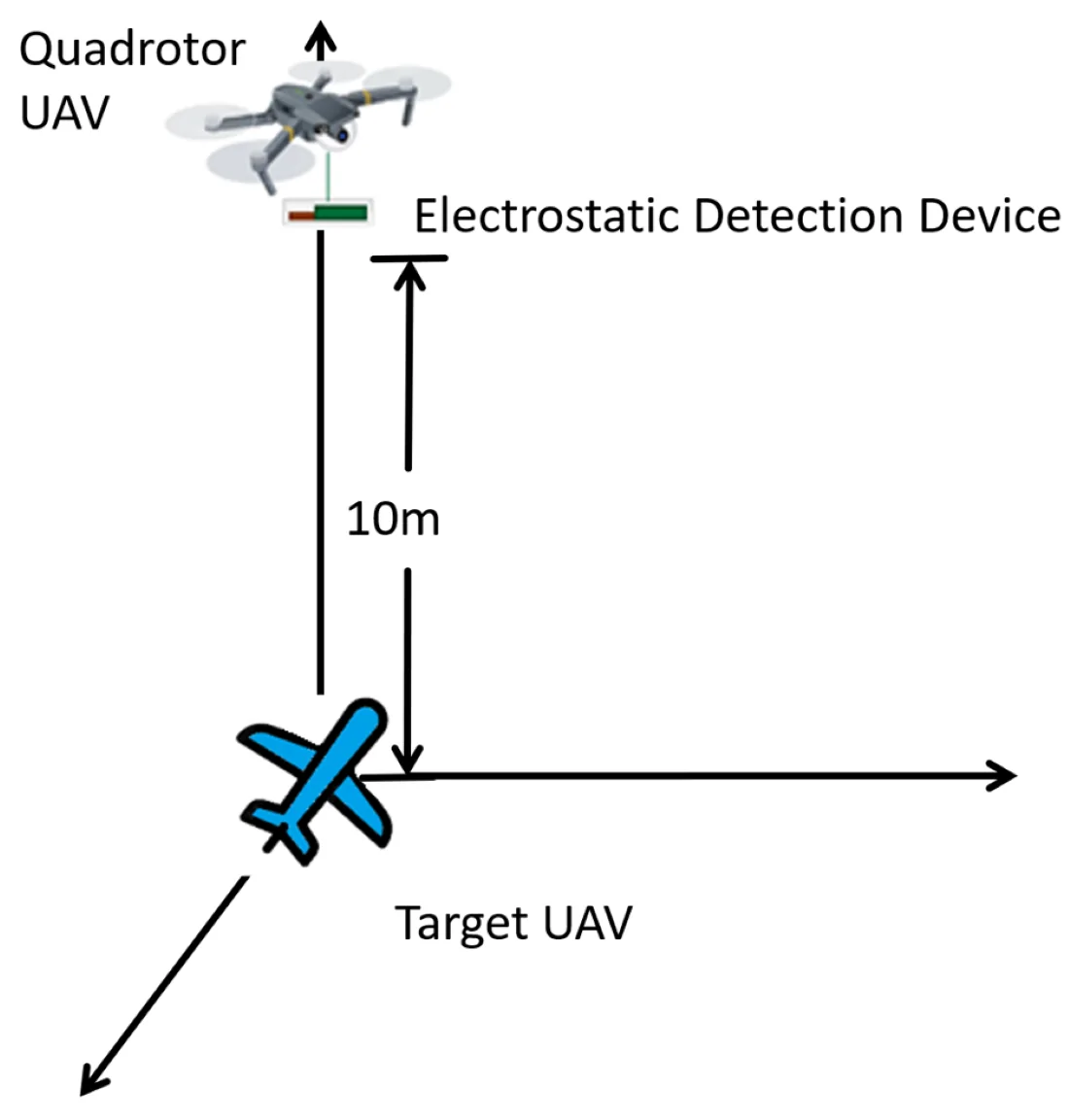
Schematic of the experimental arrangement.

To examine the agreement between the modeled and simulated response trends and those observed in flight, the experiment shown in Figure 12 was designed. A quadrotor UAV carried the electrostatic detection system to a prescribed altitude and remained approximately stationary relative to the ground. The carbon-fiber composite UAV flew horizontally 10 m below the detection system at a speed of 30 m/s and an angle of attack of 0°.

**Figure 13:**
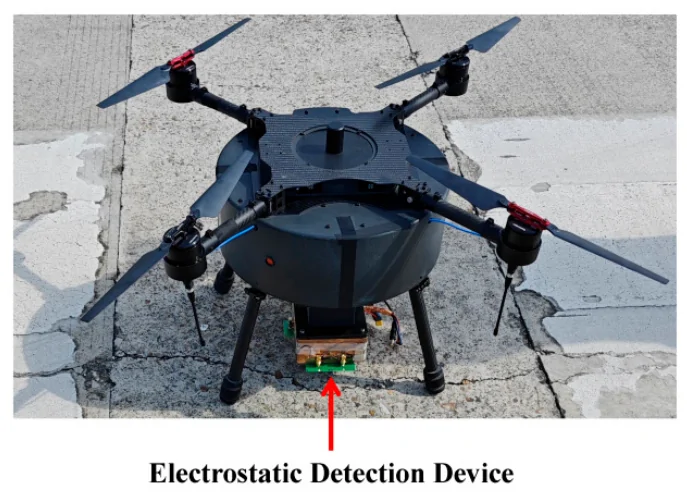
Electrostatic detection system mounted beneath the quadrotor UAV.

The electrostatic detection system was mounted beneath the quadrotor, with the sensing electrode parallel to the ground and facing the carbon-fiber composite UAV under test, as shown in Figure 13. Before the experiment, the operating status of the sensor and acquisition system was checked, and the device surface was cleaned with anhydrous ethanol to remove solid contaminants and residual charge.

**Figure 14:**
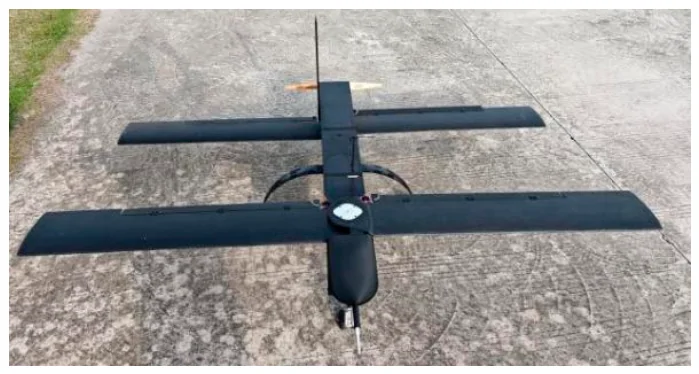
Carbon-fiber composite UAV used in the flight test.

The carbon-fiber composite UAV used in the experiment is shown in Figure 14. Its fuselage length was approximately 0.7 m and its wingspan approximately 1 m, closely matching the dimensions of the simplified model in Section 3.1.1. The surface material was carbon-fiber composite, consistent with the material specified in the simulation.

### 4.3. Experimental Results and Analysis

**Figure 15:**
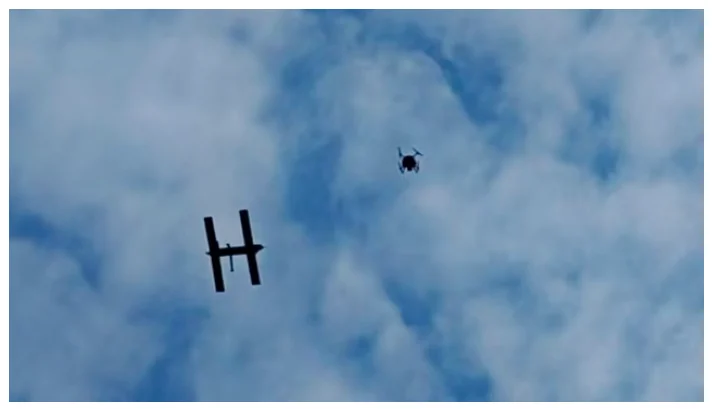
Encounter between the carbon-fiber composite UAV and the electrostatic detection system.

The encounter between the detection system and the target UAV during the experiment is shown in Figure 15.

**Figure 16:**
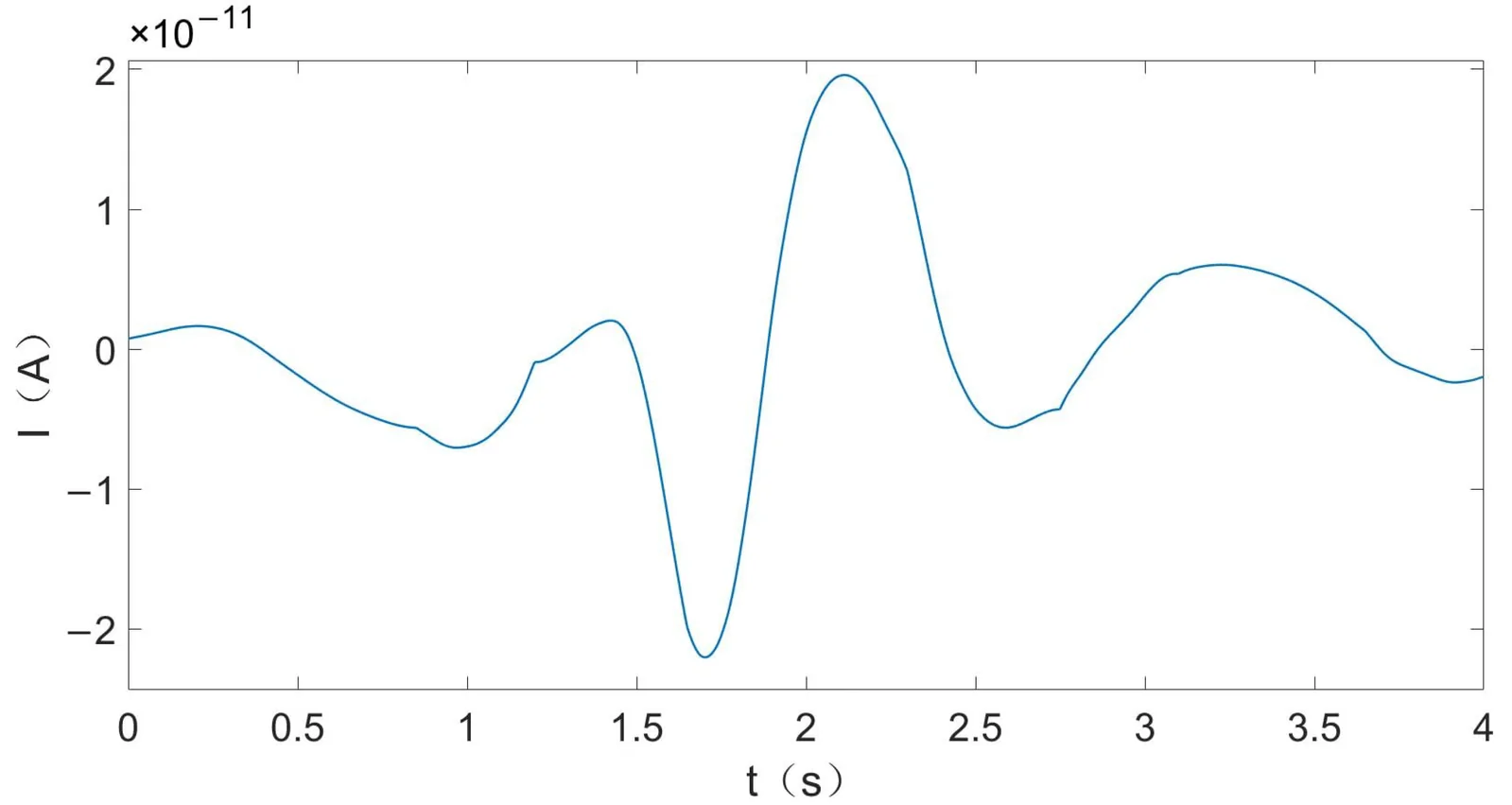
Induced current I as a function of time t at a vertical distance of 10 m and a flight speed of 30 m/s.

When the carbon-fiber composite UAV flew horizontally 10 m below the electrostatic detection system at 30 m/s, the current waveform induced on the sensing electrode was as shown in Figure 16. During the encounter, the electric-field variation caused by the changing relative position of the target and sensing electrode is a slowly varying process; the corresponding induced-current signal is therefore concentrated mainly at low frequencies. Environmental background noise, carrier-UAV vibration, and power-frequency interference can include higher-frequency components that are reduced by low-pass filtering. Background variations within the response band are considered in Section 5.3. Accordingly, a second-order Butterworth low-pass filter followed by Savitzky–Golay smoothing was applied to suppress out-of-band noise and residual fluctuations.

The induced-current signal was sampled at 1 kHz and converted from the recorded ADC counts using a ±10 V input range. A second-order causal Butterworth low-pass filter with a 2.5 Hz cutoff was applied, followed by a 500-sample (0.5 s) Savitzky–Golay smoothing step. Additional local smoothing was performed over specified sample intervals. The current was converted to dE_n_/dt using a 10 GΩ sampling resistance, an electrode area of 0.0023 m^2^, and a geometric factor of 1 + K = 1.02. The simulated curve was aligned by its minimum; it was linearly interpolated to the experimental time grid.

To examine this extraction process, one representative encounter with a stable trajectory and a complete synchronized record was selected and processed using the same procedure. The extracted waveform followed the independently obtained simulation result, supporting agreement between the processed encounter response and the simulation.

**Figure 17:**
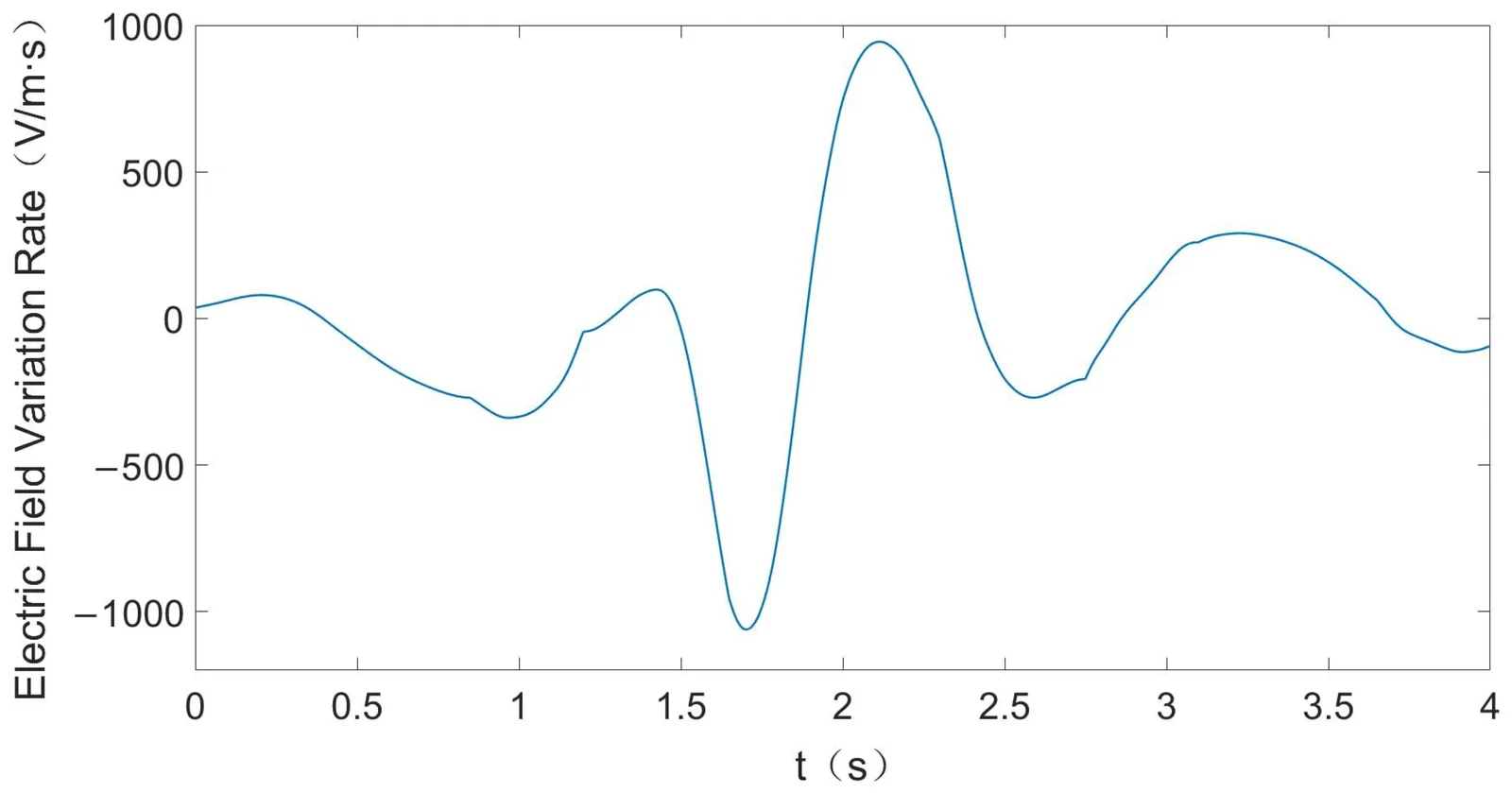
Rate of change of the electrode-normal electric-field component, dE_n_/dt, as a function of time t at a vertical distance of 10 m and a flight speed of 30 m/s.

According to the induced-current relation in Equation (17), the induced current is proportional to the time derivative of the electric-field component normal to the sensing electrode. The two signals therefore have the same temporal waveform and differ only by a scale factor. The inferred electric-field rate of change is shown in Figure 17, with a peak magnitude of approximately 1061 V/(m·s).

**Figure 18:**
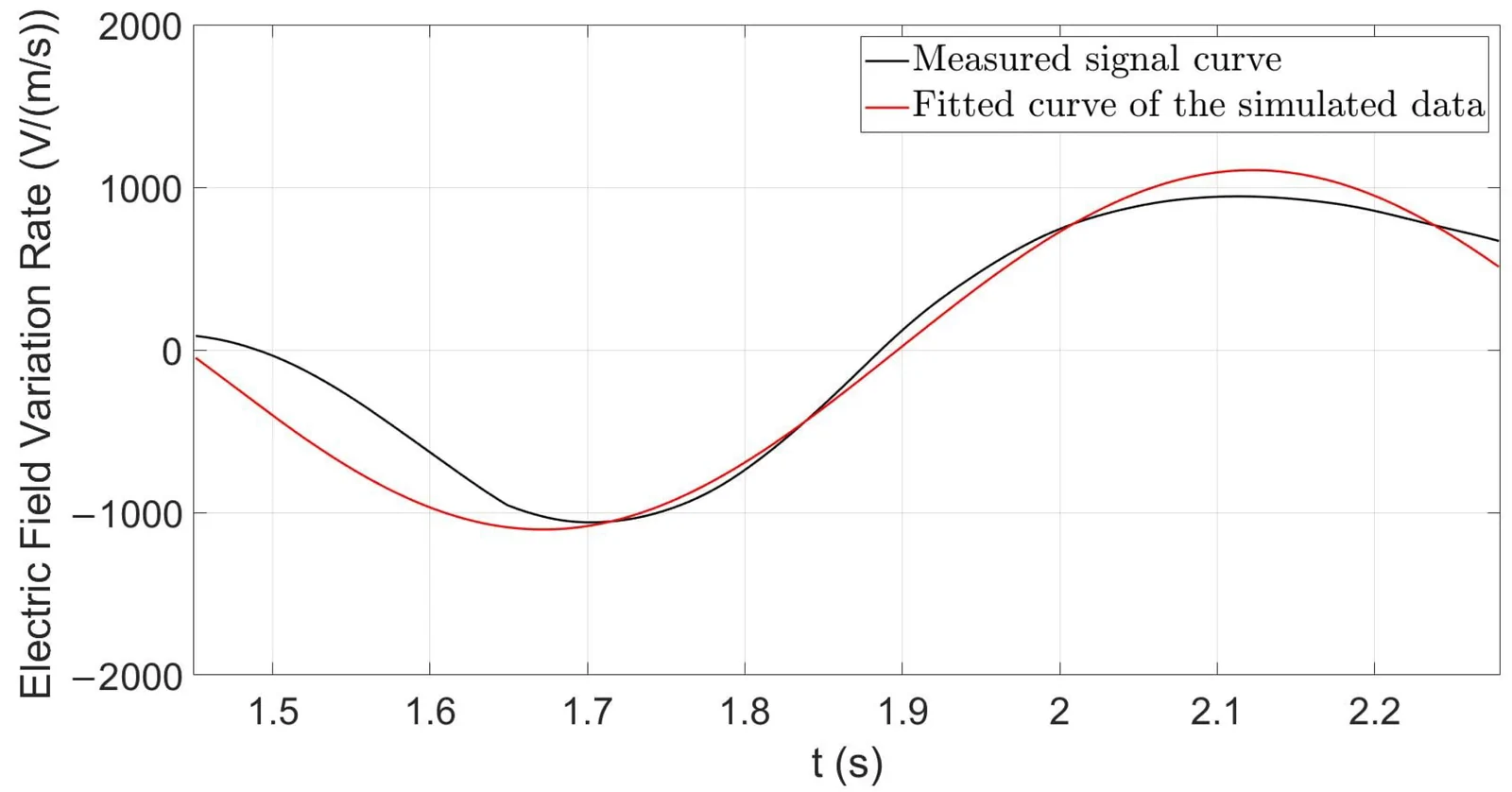
Comparison of measured (black) and simulated (red) dE_n_/dt for the selected 10 m encounter after the stated processing and time alignment.

The fitted simulation function for the electric-field rate of change was compared with the measured curve. Because the measured signal contained an initial interval with no marked variation, whereas the simulated signal represented only the principal response, their starting points differed. The signals were therefore temporally aligned by their minima, and the effective response interval was extracted for quantitative comparison. The comparison yielded an adjusted R^2^ of 0.980, indicating close agreement, as shown in Figure 18.

**Figure 19:**
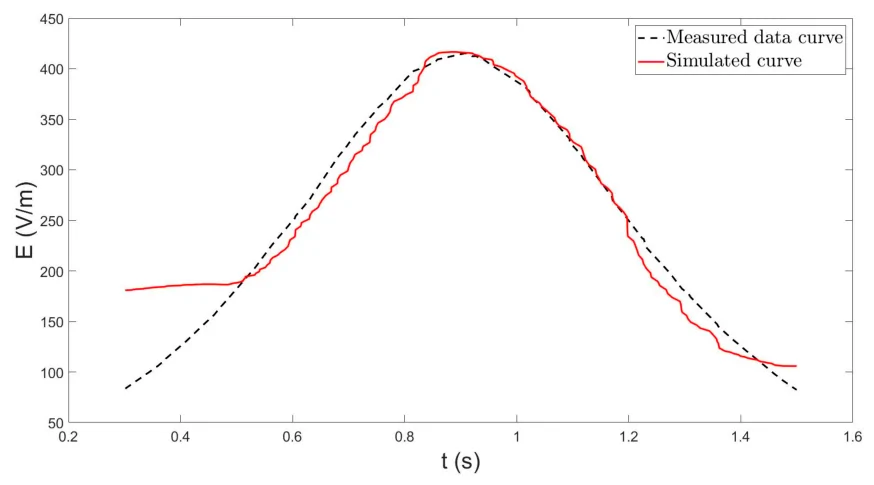
Comparison of the measured (black dashed) and simulated (red solid) electric-field strength at a vertical separation of 10 m.

The electric-field strength measured by the MEMS sensor was filtered and compared with the simulated data. The two curves had a shape-consistency coefficient of 0.937 and exhibited close agreement, as shown in Figure 19.

During actual flight, unstable airflow, small attitude variations, and fluctuations in the distribution of suspended particles prevent the gas-molecule number density and local surface flow field from remaining strictly constant. These factors introduce discrepancies between the measured, theoretical, and simulated results. Nevertheless, at a vertical distance of 10 m, a flight speed of 30 m/s, and an angle of attack of 0°, the measured and simulated electric-field rates of change had an adjusted coefficient of determination (adjusted R^2^) = 0.980, and the measured and simulated electric-field strengths had a shape-consistency coefficient of 0.937. These high levels of agreement indicate that the theoretical charging model and corresponding simulation method can characterize the variation in the near-field electric field of a small UAV under the tested condition.

Under the foregoing experimental condition, the electrostatic detector produced an identifiable induced-current response corresponding to the target encounter. The inferred electric-field rate of change had a peak magnitude of approximately 1061 V/(m·s), and its waveform was consistent with the simulated result, demonstrating that the detector responds effectively during the target encounter.

## 5. Discussion

### 5.1. Comparison Between the Electrostatic Detector and Electric-Field Sensor

Comparison of the measurements shows that the electric-field rate of change obtained with the electrostatic detector agreed more closely with the simulation than did the electric-field-strength signal. The induced-current detector inherently responds to temporal variation in the electric field at the sensing electrode and therefore more directly reflects the variation caused by the changing relative position of the target and electrode. By contrast, the absolute field measured by a conventional electric-field sensor includes contributions from the atmospheric electric field and other unknown charged sources. In this encounter, the induced-current response more closely followed the simulated trend; this suggests a potential advantage for rapidly varying target fields. With a stable electrode position and orientation, it can also suppress quasi-static background-field interference to some extent and may provide a basis for subsequent target recognition.

### 5.2. Detection Distance and Signal-to-Noise Ratio

At an encounter distance of 10 m, the target-induced electric-field variation at the sensing electrode was weak, and extraction of the target response required the band-limited processing described above. For a fixed charge distribution in free space and at distances large compared with target size, the electric field decreases approximately as r^−2^ when the net charge dominates, or as r^−3^ when the dipole term dominates [11]. Along a flyby at a fixed speed, the rate of field change decreases more steeply with distance. The response also depends on target charge, sensor orientation, electrode area, front-end sensitivity, and background fields. Accordingly, 10 m is the encounter distance at which a response consistent with the simulation was obtained using the present device and signal-processing procedure; it is not the limiting detection range of the system. Extending the effective range and improving the signal-to-noise ratio will require a larger effective sensing-electrode area, higher preamplifier sensitivity, and improved suppression of background interference.

### 5.3. Applicability and Limitations

The present results characterize a carbon-fiber composite UAV near the calibrated flight speed under the specified temperature, humidity, and dilute-particle conditions. Material structure and humidity can influence charge retention: resin-rich layers affect the electrical behavior of carbon-fiber composites [27], and relative humidity affects surface-charge decay on epoxy materials [28]. Charge redistribution within a material and loss of its total charge are different processes: an electrically isolated conductor can retain net surface charge [29]. Charge retention therefore depends on both the material and its electrical connection to the surroundings. In this experiment, the coupon was insulated from its support, and *k*(*t*) describes the net charge change during continued airflow. The local charge distribution and its decay would require specimen-specific conductivity and leakage measurements. Further studies could examine charge relaxation, varying dust and rainfall conditions, detection distance, and suppression of background interference.

The atmospheric potential gradient discussed by Feynman et al. corresponds to an electric field, with a typical near-ground fair-weather value of about 100 V/m [30]. Equation (17) describes the time derivative of the field component normal to the electrode. A constant background field produces zero induced current in this ideal relation when the electrode position and orientation are fixed. Thus, the presence of an atmospheric potential gradient alone does not establish that the measured encounter response is environmental noise. In flight, atmospheric variations, movement through field gradients, changes in attitude, and charge on the carrier UAV can contribute to the measured signal; such effects are also relevant to small-UAS atmospheric measurements [31]. An approaching target may also distort the ambient field. The agreement with the simulated waveform supports an encounter-related response under the tested conditions, while separating these contributions from the target charging response would require additional background measurements.

## 6. Conclusions

To address the need for near-field detection of small UAVs, this study proposed an electrostatic near-field detection method based on the field generated by triboelectric charging during flight and systematically analyzed the electrostatic near-field characteristics of a small UAV. A physical model linking flow-field parameters to target charge and then to electrostatic characteristics was established to support UAV detection and future target recognition research. Gas–solid two-phase simulations were used to analyze charging associated with air and low-altitude suspended particles, and fluid–electrostatic multiphysics coupling was used to determine the electrostatic characteristics of the UAV and surrounding space. UAV electrostatic detection experiments provided support for applying the charging model under the investigated condition. During a selected 10 m flight encounter, the measured and simulated electric-field rates of change had an adjusted coefficient of determination (adjusted R^2^) = 0.980, while the electric-field-strength curves had a shape-consistency coefficient of 0.937. These findings support the feasibility of the proposed near-field detection method and the model’s ability to reproduce the measured response trend under the tested conditions.

Compared with conventional radio-frequency, electro-optical, and acoustic methods, the proposed method does not depend on target electromagnetic emissions, external illumination, or acoustic signatures. It is passive and does not require an external radiation source. Moreover, because the induced-current configuration responds to the temporal rate of change of the electric field, it has the potential to suppress quasi-static background-field interference and may complement other methods in near-field scenarios involving rapid target–detector encounters. Extension to other UAV surface materials requires further calibration.

## Figures and Tables

**Table 1:** Experimental instruments and specifications.

Instrument	Model	Key Specifications	Manufacturer, City, and Country
Electrometer	Keithley 6517B	Charge range: 1 fC–2 μC; current resolution: 10 aA; input impedance: >200 TΩ in parallel with 20 pF; input bias current: <3 fA; input bias-current noise: 0.75 fA (peak-to-peak)	Keithley Instruments, Inc., Cleveland, OH, USA
Precision balance	Puruisi YP203	Capacity: 200 g; readability: 0.001 g; RS-232 output; tare function	Pruiste (Anhui) Measuring Technology Co., Ltd., Bengbu, China
Flowmeter	—	Range: 10–100 L/min	Hongcheng Electrical Appliances Co., Ltd., Suzhou, China
Synthetic-air cylinder	—	Volume: 40 L; working pressure: 15 MPa; synthetic-air purity: 99%	Huayu Tongfang Chemical Technology Development Co., Ltd., Beijing, China
Shielded enclosure	—	Acrylic glove box lined internally with 200-mesh copper screen	custom-built in-house
Specimen	Carbon-fiber composite	40 mm × 40 mm; area: 0.0016 m^2^	prepared in-house
Gas line	—	Inner diameter: 8 mm; PVC tubing and grounded stainless-steel tubing	custom-built in-house
Ambient conditions	—	Temperature: 25 °C; relative humidity: 19.6%	

**Table 2:** Dimensions of the UAV model.

Component	Length (mm)	Width (mm)	Height (mm)
Fuselage	670	140	140
Front wing	1000	100	10
Rear wing	800	100	10

**Table 3:** UAV triboelectric charging characteristics at different angles of attack.

Angle of Attack	Maximum Surface Charge Density (C/m^2^)	Minimum Surface Charge Density (C/m^2^)	Total Triboelectric Charge (C)
0°	7.804 × 10^−7^	6.790 × 10^−9^	7.652 × 10^−7^
10°	7.650 × 10^−7^	6.686 × 10^−9^	7.534 × 10^−7^
15°	7.543 × 10^−7^	7.174 × 10^−9^	7.469 × 10^−7^
20°	7.433 × 10^−7^	6.725 × 10^−9^	7.363 × 10^−7^

## Data Availability

The experimental data supporting the conclusions of this article are internal laboratory data and are not publicly available because of laboratory data-management requirements and restrictions concerning technical information about the apparatus. The data are available from the corresponding author upon reasonable request and subject to approval by the laboratory.

## Abbreviations

The following abbreviations are used in this manuscript:

UAV	Unmanned Aerial Vehicle
MEMS	Microelectromechanical systems

## References

[1] Seidaliyeva U., Ilipbayeva L., Taissariyeva K., Smailov N., Matson E.T. (2024). Advances and challenges in drone detection and classification techniques: A state-of-the-art review. Sensors.

[2] Jiang W., Liu Z., Wang Y., Lin Y., Li Y., Bi F. (2024). Realizing small UAV targets recognition via multi-dimensional feature fusion of high-resolution radar. Remote Sens..

[3] Vitiello F., Causa F., Opromolla R., Fasano G. (2024). Radar/visual fusion with fuse-before-track strategy for low altitude non-cooperative sense and avoid. Aerosp. Sci. Technol..

[4] Zhao L., Zhang Y., Li Y., Zhong H. (2025). Global and local context-aware detection for infrared small UAV targets. Drones.

[5] Munir A., Siddiqui A.J., Anwar S., El-Maleh A., Khan A.H., Rehman A. (2024). Impact of adverse weather and image distortions on vision-based UAV detection: A performance evaluation of deep learning models. Drones.

[6] Yasmeen A., Daescu O. (2025). Recent research progress on ground-to-air vision-based anti-UAV detection and tracking methodologies: A review. Drones.

[7] Kyritsis A., Makri R., Uzunoglu N. (2022). Small UAS online audio DOA estimation and real-time identification using machine learning. Sensors.

[8] Liu Z.R., Fan K.G., Chen Y.H., Xiong L.Z., Ye J.Z., Fan A.G., Zhang H.H. (2025). Deep learning-based acoustic recognition of UAVs in complex environments. Drones.

[9] Semenyuk V., Kurmashev I., Lupidi A., Alyoshin D., Kurmasheva L., Cantelli-Forti A. (2025). Advances in UAV detection: Integrating multi-sensor systems and AI for enhanced accuracy and efficiency. Int. J. Crit. Infrastruct. Prot..

[10] Famili A., Stavrou A., Wang H., Park J.-M., Gerdes R. (2024). Securing your airspace: Detection of drones trespassing protected areas. Sensors.

[11] Chen X., Li P.F., Tang K., Cui Z.Z. (2019). Principles and Applications of Electrostatic Detection.

[12] Qi H.B. (2023). Research on Electrostatic Detection Method of Quadrotor UAV Swarms. Master’s Thesis.

[13] Li Z.Y. (2023). Simulation and Detection Methods for the Near-Field Electrostatic Characteristics of Large Aircraft. Master’s Thesis.

[14] Wang G., Yang P., Chu Z., Ran L., Li J., Zhang B., Wen X. (2024). A review on resonant MEMS electric-field sensors. Micromachines.

[15] Jensen P.T. (2021). Practical Measurement of Aircraft Electrostatic Charging. https://resourcecenter.ieee.org/conferences/emcsipi-2021/emcsipi20213350.

[16] Sun L., Wang Z., Li C., Tang W., Wang Z. (2023). Probing Contact Electrification between Gas and Solid Surface. Nanoenergy Adv..

[17] Lu S., Cheng Z., Long W., Xu X., Qian H. (2025). Contact electrification at gas-solid interface via pulsed gas jets. Cell Rep. Phys. Sci..

[18] Sun L.L. (2022). Charging Mechanism and Electrostatic Damage of Near-Space Long-Endurance Solar-Powered UAVs. Master’s Thesis.

[19] Tao R.L., Wang Z.H. (2024). Gas-surface interaction features under effects of gas-gas molecules interaction in high-speed flows. Chin. J. Aeronaut..

[20] Chen D.D., Wang B.W. (2020). Fundamentals of Engineering Fluid Mechanics.

[21] Zhao S.W., Wang C.Z., Yang J., Li W., Zhang Y.K., Wang P.P. (2014). Difference analysis of gas equations of state applied in plateau environments. Meteorol. Hydrol. Mar. Instrum..

[22] Anderson J.D. (2010). Fundamentals of Aerodynamics.

[23] Matsusaka S., Maruyama H., Matsuyama T., Ghadiri M. (2010). Triboelectric charging of powders: A review. Chem. Eng. Sci..

[24] Liu S.H., Xie X.N., Hu X.F. (2021). Research progress on electrostatic generation and discharge of aircraft. Saf. Electromagn. Compat..

[25] Wang X., Wen H., Shi J., Bi J., Huang Z., Zhang B., Zhou T., Fu K., Chen Q., Xin J. (2018). Optical and microphysical properties of natural mineral dust and anthropogenic soil dust near dust source regions over northwestern China. Atmos. Chem. Phys..

[26] Ryder C.L., Marenco F., Brooke J.K., Estelles V., Cotton R., Formenti P., McQuaid J.B., Price H., Liu D., Rosenberg P. (2018). Coarse-mode mineral dust size distributions, composition and optical properties from AER-D aircraft measurements over the tropical eastern Atlantic. Atmos. Chem. Phys..

[27] Yu H., Advani S., Heider D. (2019). Impact of resin-rich layer on the through-thickness resistivity of carbon fiber reinforced polymers. J. Compos. Mater..

[28] Lutz B., Kindersberger J. Influence of relative humidity on surface charge decay on epoxy resin insulators. Proceedings of the 2009 IEEE 9th International Conference on the Properties and Applications of Dielectric Materials.

[29] Feynman R.P., Leighton R.B., Sands M. The Feynman Lectures on Physics, Vol. II; Chapter 5, Application of Gauss’ Law, Section 5–9. California Institute of Technology Online Edition. https://www.feynmanlectures.caltech.edu/II_05.html.

[30] Feynman R.P., Leighton R.B., Sands M. The Feynman Lectures on Physics, Vol. II; Chapter 9, Electricity in the Atmosphere, Section 9–1. California Institute of Technology Online Edition. https://www.feynmanlectures.caltech.edu/II_09.html.

[31] Schön M., Nicoll K.A., Büchau Y.G., Chindea S., Platis A., Bange J. (2022). Fair-weather atmospheric charge measurements with a small UAS. J. Atmos. Ocean. Technol..

